# Infra-patellar fat pad-derived mesenchymal stem cells maintain their chondrogenic differentiation potential after arthroscopic harvest with blood-product supplementation

**DOI:** 10.1007/s00264-023-05930-7

**Published:** 2023-08-30

**Authors:** Markus Neubauer, Alexander Otahal, Olga Kuten, Seth Lawrence Sherman, Lukas Moser, Karina Kramer, Andrea DeLuna, Johannes Neugebauer, Dietmar Dammerer, Thomas Muellner, Stefan Nehrer

**Affiliations:** 1 Center for Regenerative Medicine and Orthopaedics, Danube University Krems, Dr. Karl-Dorrek-Str. 30, 3500 Krems, Austria; 2 Division of Orthopaedics and Traumatology, University Hospital Krems, Karl Landsteiner University of Health Sciences, Dr. Karl-Dorrek-Straße 30, 3500 Krems, Austria; 3 Ortho Sera GmbH, Dr. Karl-Dorrek-Str. 30, 3500 Krems, Austria; 4 Department of Orthopaedic Surgery, Stanford University, Redwood City, CA USA; 5 Department of Orthopaedics and Traumatology, Evangelic Hospital Vienna, Hans-Sachs-Gasse 10–12, 1180 Vienna, Austria

**Keywords:** Mesenchymal stem cells, Infrapatellar fat pad, Blood products, Cartilage regeneration

## Abstract

**Purpose:**

Mesenchymal stem cells/medicinal signaling cells (MSCs) possess therapeutic potential and are used in regenerative orthopaedics. The infra-patellar fat pad (IFP) is partially resected during knee arthroscopy (KASC) and contains MSCs. Heat, irrigation, and mechanical stress during KASC may decrease MSC’s therapeutic potential. This study assessed MSCs’ regenerative potential after arthroscopic IFP harvest and potential effects of two blood products (BP) (platelet-rich plasma (PRP), hyperacute serum (HAS)) on MSCs’ viability and chondrogenic differentiation capacity.

**Methods:**

IFP was arthroscopically harvested, isolated, and counted (*n* = 5). Flow cytometry was used to assess cell viability via staining with annexin V/7-AAD and stemness markers via staining for CD90, CD73, and CD105. MSCs were incubated with blood products, and metabolic activity was determined via an XTT assay. Deposition of cartilage extracellular matrix was determined in histologic sections of chondrogenically differentiated 3D pellet cultures via staining with Alcian Blue. Expression of cartilage-specific genes (SOX9, MMP3/13, ACAN, COL1/2) was analyzed via quantitative PCR.

**Results:**

MSC isolation from IFP yielded 2.66*10^6^ ± 1.49*10^6^ viable cells from 2.7 (0.748) g of tissue. MSC markers (CD 90/105/73) were successfully detected and annexin V staining showed 81.5% viable cells. XTT showed increased metabolic activity. Within the BP groups, this increase was significant (days 0–14, *p* < 0.05). PCR showed expression of cartilage-specific genes in each group. COL2 (*p* < 0.01) as well as ACAN (*p* < 0.001) expression levels were significantly higher in the HAS group. Histology showed successful differentiation.

**Conclusion:**

Arthroscopic harvest of IFP-MSCs yields sufficient cells with maintained regenerative potential and viability. Blood products further enhance MSCs’ viability.

## Introduction

Preventing and treating OA, a common and debilitating disease with an increasing incidence correlated to age, is a major goal of orthopaedics warranting novel and individualized treatment options [1].

In regenerative orthopaedics, research and clinical use of mesenchymal stem cells/medicinal signaling cells (MSCs) as a novel cell-based treatment is rapidly growing [2]. MSCs do have the potential to (i) treat osteoarthritis (OA) predominantly by altering joint homeostasis towards a decreased inflammatory environment—a major driver of further cartilage degeneration and (ii) foster joint preservation as a cell source for cartilage regeneration [3, 4]. Thus, MSCs can be used for cartilage tissue engineering to treat (osteo-) chondral lesions (OCL)—that contribute to OA development [5]—as well as a disease-altering intervention for manifest OA [3, 4].

Key features mediating MSCs’ effects are their paracrine factors triggering immunomodulation, anti-inflammation, and others. The cells’ differentiation potential into chondrocytes, osteocytes, and other cells may play a role especially for cartilage tissue engineering [6–8]. However, this “engrafting” is not considered the primary mode of action in the current literature.

**Fig. 1:**
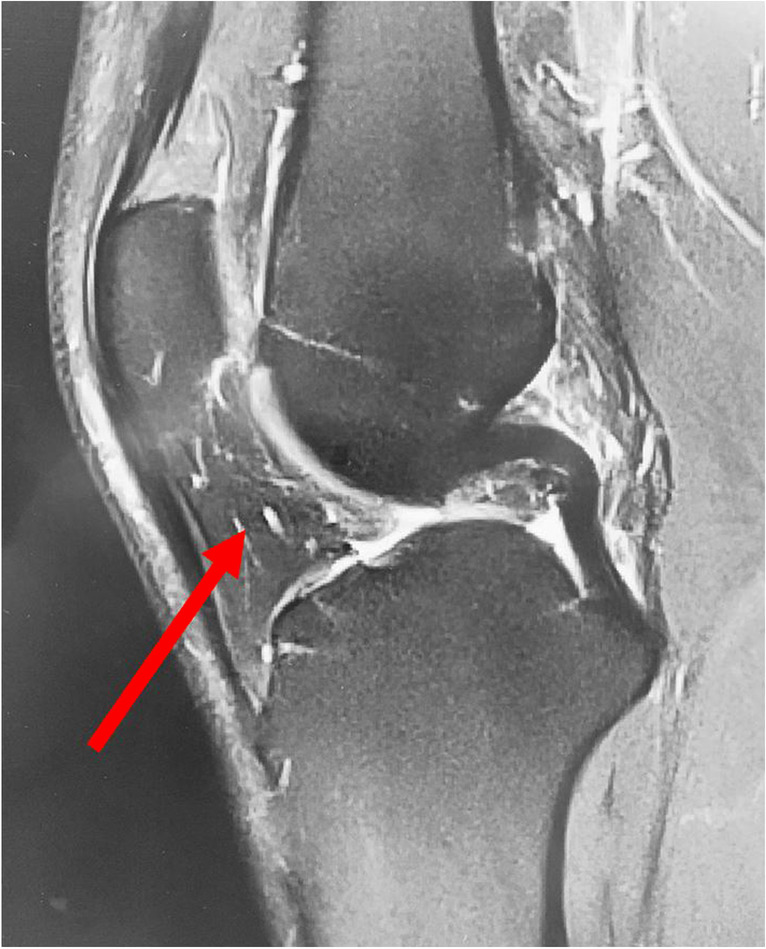
IFP–hypertrophy (marked with a red arrow): anonymized picture of an actual donor—taken pre-surgically

The infra-patellar fat pad (IFP), an anatomical structure that lies intra-articularly in the knee but extra-synovially, is becoming an increasingly prominent adipose MSC source in comparison to commonly used subcutaneous adipose tissue [9]. The benefits of IFP-MSCs are as follows: (i) less regulatory hurdles, (ii) superior biological features, and (iii) lower harvest morbidity [9]. (i) IFP-MSCs were shown to possess superior chondrogenic differentiation potential. Moreover, the IFP itself has been shown to be a reservoir of disease-alerting cellular products that do play a role in the physiological joint homeostasis [9–11].

(ii) IFP resection during arthroscopy has been shown to be easy, safe, and beneficial for pain relief and function in case of IFP hypertrophy (Fig. 1) [12, 13]. The IFP is easily accessible during arthroscopy, and IFP remnants are usually discarded as surgical waste. Figure 1 shows a hypertrophic IFP in an MRI picture.

Controversy regarding the usability of arthroscopically harvested IFP-MSCs warrants further investigation of biological features. Arthroscopic harvest may decrease MSCs’ viability due to mechanical stress, including shear or frictional heat.

Cellular adaptive mechanisms might be triggered by thermal stress resulting in changes in gene expression and protein function [14, 15]. Arthroscopic procedures can expose tissue to substantial thermal stress [16].

In cadaveric models, it was shown that temperature increases up to 52.0 °C during arthroscopy with radiofrequency application and irrigation [17]. Exposure of MSC to arthroscopy typical heat was reported to alter cells’ metabolic activity and also upregulate heat shock-related genes [18].

Autologous blood products (BP) such as platelet-rich plasma or hyperacute serum (HAS)—a novel BP that mimics the coagulation cascade—were shown to have beneficial effects upon IFP-MSC biological features such as their metabolic activity and their regenerative potential [19].

Also, BPs are an emerging therapeutic option for OA [20–23], and the administration of MSCs supplemented with blood products was shown to have beneficial effects in painful degenerative knees [23, 24].

A synergistic beneficial effect of BP and IFP-MSCs for cells’ biological features is likely, warranting data to support this rationale.

Different biological features and formulations of cells and orthobiologic therapies, in general, are likely associated with variations in clinical outcomes [25–27]. The process of translation of biological therapies in musculoskeletal medicine largely depends on the precise collection of biological characterization data correlated with clinical outcomes in registries as stated by Chu et al. [28].

The purpose of this study was to contribute data to enlarge biological knowledge about IFP-MSCs after arthroscopic harvest and effects of blood product supplementation on IFP-MSCs regarding viability and their chondrogenic differentiation potential.

## Materials and methods

### Tissue harvest

The IFP was arthroscopically harvested from five donors (*n* = 5, mean age: 54 ± 2,3 a; w:m = 3:2; Kellgren Lawrence OA score: 1–2). Four donors were scoped due to a locking joint accompanied by a degenerative tear. One donor was scoped due to a plica syndrome without a meniscal tear. Signed informed consent was received from each donor (Commission for Scientific Integrity and Ethics Karl Landsteiner University Krems EC Nr: 1022/2020).

During arthroscopic harvest, a total resection of the IFP was avoided. The shaving resulted in mincing of the tissue in the course of the procedure, and the tissue fragments were collected in a sieve (Storz, Drillcut-Arthro-Shaver, oscillating, 3000 rounds/min, aggressive full radius resector 3.5 mm).

The harvested tissue was then stored in PBS at + 8°. Temperature was monitored during storage and transport to the cell culture lab, which was done within 24 h.

### Cell isolation and culture

First, fragmented tissue was weighed. Secondly, the tissue was enzymatically digested by adding collagenase (#C0130. Sigma-Aldrich, St. Louis, USA): 9000 units of collagenase I/15 ml DMEM/10 g of fat. Afterwards, the mixture was placed in an incubator for 2 h at 37 °C on a shaking machine with 25 rpm to optimize the tissue digestion and cell release.

After digestion, the suspension was filtered using a 40 μm cell strainer (Corning® 40 μm cell strainer, #431750. Durham, USA). The cells were washed once with 10 ml PBS via centrifugation and resuspended in MSC growth medium to neutralize remaining collagenase I (DMEM, high glucose, GlutaMAXTM Supplement, pyruvate, #31966047, Gibco Life Technologies Europe Bv, Bleiswijk, Netherlands) with added antibiotics (2% penicillin/streptomycin (#P4333, Sigma-Aldrich Chemie GmbH, Steinheim, Germany) and 1% amphotericin B (#A2942, Sigma-Aldrich Chemie GmbH, Steinheim, Germany)). Moreover, the medium contained 10% fetal calf serum (#11550356, Gibco, qualified, heat inactivated, Gibco Life Technologies Europe Bv, Bleiswijk, Netherlands), 1% non-essential amino acids (MEM NEAA #11140050. Gibco Life Technologies Europe Bv, Bleiswijk, Netherlands), and bFGF 1 ng/mL (Fibroblast Growth Factor-Basic, human, #F0291, Sigma-Aldrich Chemie GmbH, Steinheim, Germany).

Cells were seeded at a density of 10^4^ cells/cm^2^ in T175 cell culture flasks and grown until a confluency of 80–90%. Nonadherent cells were discarded with the first MSC growth medium change after 24 h, and then medium was changed every two to three days. For passaging, cells were enzymatically detached by 1 ml undiluted accutase (#SCR005, Sigma- Aldrich Chemie GmbH, Steinheim, Germany) per flask.

### Preparation of blood products

#### Platelet-rich plasma (PRP)

Whole blood was drawn from donors using standard blood collection tubes containing trinatriumcitrate as an anticoagulant (VACUETTE #455322; Greiner Bio-One, Kremsmünster, Austria).

The citrate-anticoagulated PRP (CPRP) preparation procedure involved four steps:

1. Centrifugation of the whole blood at 440 × g for ten min at room temperature. Thereby, three layers are formed in the test tube: (i) a bottom layer—predominantly consisting of red blood cells, (ii) a middle layer—containing buffy coat with leucocytes, and (iii) top layer—containing platelets and plasma.
2. Transfer of the top plasma layer into 15 ml tubes.
3. Centrifugation of the transferred plasma layer at 1700 × g for ten min.
4. Resuspension of the formed platelet pellet in 50% of the remaining (platelet-poor) supernatant.

Each batch of CPRP consisted of pooled CPRP from up to 5 blood donors. CPRP was stored at − 80 °C until being used for experiments.

### Hyperacute serum

HAS was prepared as described in Kardos et al. [21]. Briefly, whole blood was drawn from healthy donors with the company’s device (#700194, OrthoSera, Krems an der Donau, Austria) according to the manufacturer’s protocol. In contrast to CPRP preparation, HAS preparation involved only one centrifugation step at 1710 × g for five min at room temperature immediately after drawing the blood. Then, coagulation proceeded in the upper device chamber for 30 min to form a platelet-rich fibrin clot separated from erythrocytes in the lower chamber. The lower chamber was removed, and HAS was squeezed from the fibrin clot in the upper chamber into a collection tube to be stored at − 80 °C until use.

### Chondrogenic differentiation

The chondrogenic differentiation medium contained DMEM with 54.5 g/L glucose, 0.11 g /L sodium pyruvate (#21969, Gibo Life Technologies), GlutaMAX Supplement (#35050061, Gibco Life Technologies), 5 ng/ml TGFbeta-3 (#AF-100-36E, PeproTech, New York, USA), 50 μg/mL ascorbic acid (#A4403, Sigma-Aldrich Chemie GmbH, Steinheim, Germany), 1% (v/v) ITS (ITS Liquid Media Supplement, #I3146, Sigma-Aldrich Chemie GmbH, Steinheim, Germany), 100 nM dexamethasone (#D4902, Sigma-Aldrich Chemie GmbH, Steinheim, Germany), 1% (v/v) non-essential amino acids (#11140050, Gibco Life Technologies Europe Bv, Bleiswijk, Netherlands), and 0.2% (v/v) methyl cellulose (Sigma-Aldrich Chemie GmbH, Steinheim, Germany) together with 10% (v/v) CPRP. The differentiation medium was supplemented with 2 U/ml heparin (#3909969, Gilvasan GmbH, Vienna, Austria) to prevent clotting of CPRP. A 3D structure was prepared by forming chondrogenic pellets. Therefore, 250,000 cells were placed in 15-ml polypropylene falcon tubes with the addition of chondrogenic medium. Pellets were then formed by centrifugation at a speed of 4164 × g for ten min. The differentiation period lasted three weeks. Change of chondrogenic media with blood products was done twice a week.

### Metabolic activity assay (XTT assay)

IFP-MSCs were seeded in 96-well plates (#260860. Thermo Fisher Scientific, Waltham, USA) at a density of 3125 per cm^2^ (1000 cells/well). Cells were incubated for 48 h in 100 μl MSC growth medium. Forty-eight hours following seeding (day 0), the standard medium was replaced with 100 μl medium supplemented with 10% of each blood product (PRP, HAS) or 10% FCS. PRP samples were additionally supplemented with 2 U/ml heparin (#3909969, Gilvasan GmbH, Vienna, Austria). Metabolic activity of the IFP-MSCs was measured by the XTT assay (#11465015001, Roche Diagnostics GmbH, Mannheim, Germany) according to the manufacturer’s protocol. Absorbance was measured on a plate reader (Synergy 2, BioTek Instruments, Inc., Vermont, USA) at 492 nm with reference wavelength 690 nm. The measurements were performed on days zero, three, seven, ten and 14. Each measurement was performed in biological triplicates with a cell-free medium control well to determine background absorbance. Data were normalized to day zero.

### Annexin V/7AAD staining

To detect early apoptotic and/or damaged cells, isolated MSCs were stained with annexin V and 7-AAD as per manufacturer’s protocol (BD Biosciences, #559763).

Cells were resuspended in binding buffer (PBS, 1% BSA, 10 mMCaCl_2_) at a concentration of 1.0 × 10^6^ cells/ml. One hundred microliters of this suspension was used per test and stained with 5 µl PE::annexin V and 5 µl/-AAD directly in the sample tube. The suspension was briefly vortexed and incubated for at least 30 min at room temperature protected from light. Finally, 300 µl of binding buffer was added. Afterwards, the suspension was analyzed via Cytoflex S cytometer, and data were visualized via FlowJo X.

### Flow cytometric analysis of MSC stemness markers

IFPs were cultured to 90% confluency in MSC growth medium, detached via accutase, and resuspended in binding buffer (PBS, 1% BSA). 10^5^ cells were used per test and were stained with antibodies directed against positive and negative markers as listed in Table 1, using the BD Stemflow hMSC Analysis Kit (BD Biosciences, St. Louis, USA).

**Table 1:** Antibodies used for flow cytometry

Tube	Antibodies
1	FITC mouse anti-human CD90 (1 μl)
3	PerCP-Cy^TM^5.5 mouse anti-human CD105 (1 μl)
4	APC mouse anti-human CD73 (1 μl)
5	Nothing
6	hMSC positive isotype control cocktail (4 μl), PE hMSC negative isotype control cocktail (4 μl)
7	hMSC positive cocktail (20 μl), PE hMSC negative cocktail (20 μl)

The suspension was briefly vortexed and incubated for at least 60 min at room temperature protected from light. Finally, 300 µl of binding buffer was added. Afterwards, the suspension was analyzed via Cytoflex S cytometer, and data were visualized via FlowJo X.

### Cell counting

Cell numbers were determined via trypan blue dye exclusion assay mixing 0.4% trypan blue solution (#T8154, Sigma-Aldrich) 1:1 with an aliquot of a cell suspension and counting in a chamber.

### RNA extraction and quantitative real-time polymerase chain reaction (qRT-PCR)

Differentiated cells were collected and pooled per group in 200 μl PBS. Then, those cells were homogenized in a MagNA Lyser device (RocheTM MagNA Lyser Benchtop Homogenization System) by adding ceramic beads (MagNA Lyser Green Beads #03358941001, Roche Diagnostics, Basel, Switzerland). The homogenization (6500 rpm, 20 s) was performed twice, with a two min break for cooling down (8–12 °C). The RNA isolation was done as per manufacturer’s protocol with the High Pure RNA Isolation Kit (#11828665001, Roche Diagnostics GmbH, Mann- heim, Germany).

cDNA synthesis was done with the Transcriptor First Strand cDNA Synthesis Kit (#04379012001, Roche, Basel, Switzer- land). RT-qPCR was done using the FastStart Essential DNA Probes Master Kit (#06402682001, Roche, Basel, Switzerland) in triplicates. One microliter of the cDNA product, FastStart Probe Master 2x, hydrolysis probe (final concentration 250 nM), and primers (final concentration of 900 nM) were used for PCR amplification on a Roche LightCycler 96. The design for each primer was intended to span exon-exon boundaries to avoid amplification of residual genomic DNA. Six genes were analyzed: collagen type 2 (COL2AB), collagen type 1 (COL1), SOX9, matrix metalloproteinase-3 (MMP3), matrix metalloproteinase-13 (MMP13), and aggrecan (ACAN) using GAPDH as reference gene. The sequences of the used primers are displayed in Table 2.

**Table 2:** Primer sequences used (ACAN, aggrecan; COL1, collagen 1A1 gene; COL2, collagen 2 gene; GAPDH, glyceraldehyde 3-phosphate dehydrogenase; MMP (1/13), matrix metalloproteinase-3(/13) gene; SOX9, SRY-box transcription factor 9)

Name	Identification	Forward	Reverse	Annealing T°
GAPDH	NM_002046	ACATCGCTCAGACACCATG	TGTAGTTGAGGTCAATGAAGGG	60
ACAN	NM_001135	TGTGGGACTGAAGTTCTTGG	AGCGAGTTGTCATGGTCTG	62
COL1A1	NM_000088	CCCCTGGAAAGAATGGAGATG	TCCAAACCACTGAAACCTCTG	62
COL2A1	NM_001844	AAGACGTGAAAGACTGCCTC	TTCTCCTTTCTGTCCCTTTGG	60
MMP3	NM_002422	CCAGGGATTAATGGAGATGCC	AGTGTTGGCTGAGTGAAAGAG	60
MMP13	NM_002427	GATGACGATGTACAAGGGATCC	ACTGGTAATGGCATCAAGGG	60
SOX9	NM_000346	ACTTGCACAACGCCGAG	CTGGTACTTGTAATCCGGGTG	60

### Histology

Chondropellets were embedded in cryosectioning medium (Tissue-Tek® O.C.T.TM, #4583, Sakura Finetek, Alphen aan den Rijn, The Netherlands) in disposable base molds ( #22–363- 554, Fisher Scientific, Hampton, USA). Then, samples were transferred to − 80 °C for at least 24 h. Then, the embedded chondropellets were sectioned on a cryostat device into 6 μm slices (Cryostar NX70. Thermo Fisher Scientific, Waltham, USA) and placed on adhesive glass (Thermo ScientificTM SuperFrost PlusTM, #10149870. Thermo Fisher Scientific, Waltham, USA). Glass slides were dried and fixed in cold acetone (− 20 °C) for ten min.

The sections were put in 1% Alcian Blue solution (#A3157, Sigma-Aldrich Chemie GmbH, Schnelldorf, Germany) for 30 min to stain glucosaminoglycans. Then, sections were washed in running tap water for 1 min and dehydrated through 95% ethanol and 2 changes of absolute ethanol, for three min each. Slides were cleared with xylene and covered with glass. Cartilage glycosaminoglycans appeared blue.

Hematoxylin (Mayer’s Hematoxylin, #S3309, DakoCytomation, Carpinteria, USA) and Eosin (#230251, Sigma-Aldrich Chemie GmbH, Schnelldorf, Germany) staining was performed according to the manufacturer’s protocol (DakoCytomation).

Micrographs of the pellet slices were taken with a light microscope (DM-1000 Microscope, Leica Microsystems) and processed using a Leica Manager software (Leica Microsystems, Wetzlar, Germany).

### Statistics

Statistical analysis was performed using GraphPad Prism 5 software. PCR and metabolic activity data results were analyzed via ANOVA combined with Tukey–Kramer’s multiple comparison posthoc test. ANOVA assumptions were verified. *p* < 0.05 was considered significant. Data were presented as mean ± SEM.

## Results

### Cell yield and IFP-MSC characterization

The mean weight of harvested IFP tissue was 2.7 $\pm$ 0.748 (SD) g. The total cell yield per donation was 2.66 $\pm$ 1.49 * 10^6^ (SD). This results in a cell yield of 0.9 ± 0.3* 10^6^ (SD) cells/g tissue. The results of the individual donors are displayed in Table 3.

**Table 3:** Weight of arthroscopically harvested tissue per donor in conjunction with the associated cell number; SD, standard deviation

Donor	Weight (g)	Cell number (in millions)	Cells per gram tissue (in millions)
1	2.5	1.4	0.6
2	2	1.7	0.8
3	3	3.1	1.0
4	4	5.4	1.4
5	2	1.7	0.9
mean $\pm$ SD	2.7 ± 0.7	2.7 ± 1.5	0.9 ± 0.3

**Fig. 2:**
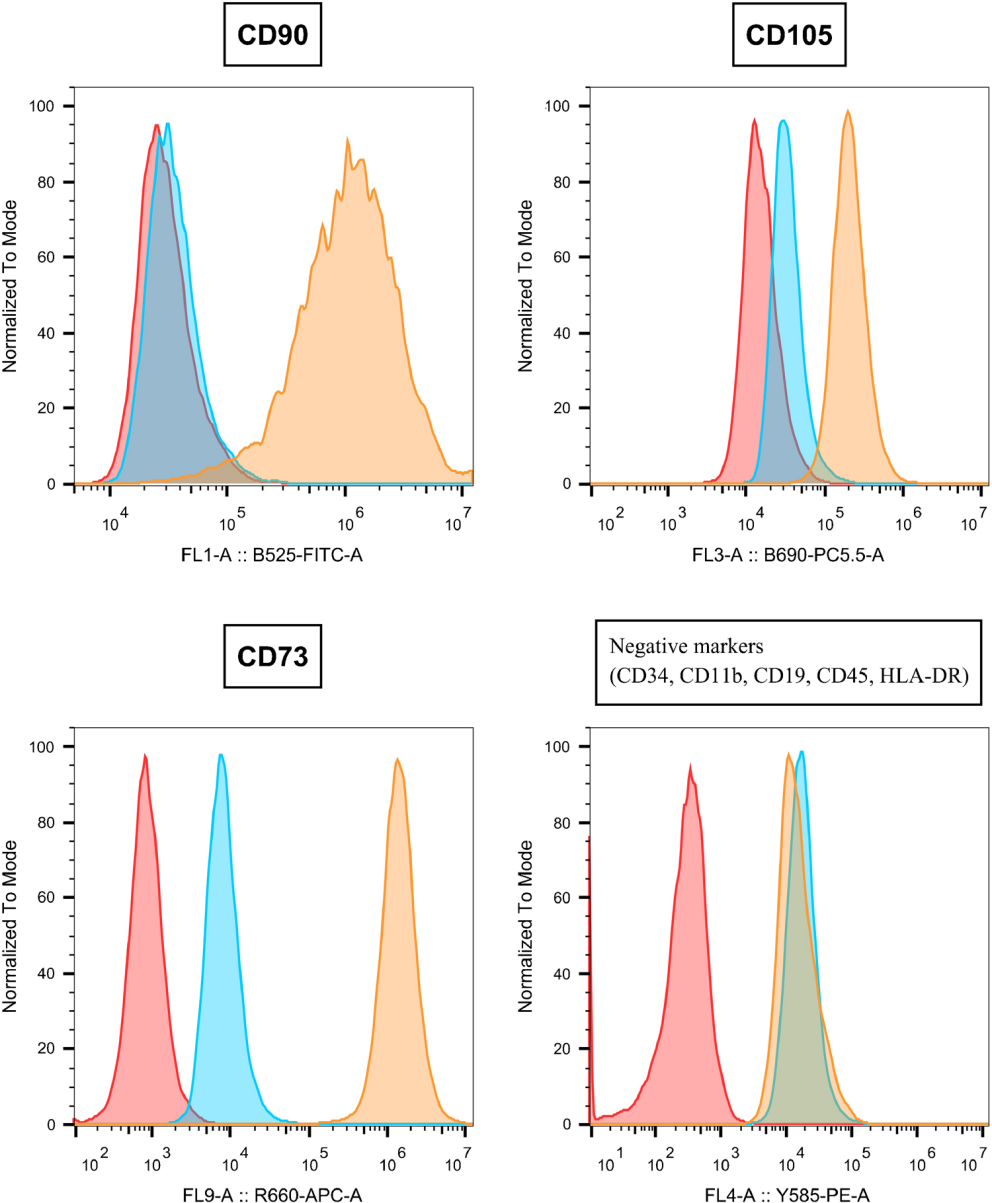
Flow cytometry: results from a representative donor: red = cells only; orange = marker; blue = isotype; CD, cluster of differentiation

**Fig. 3:**
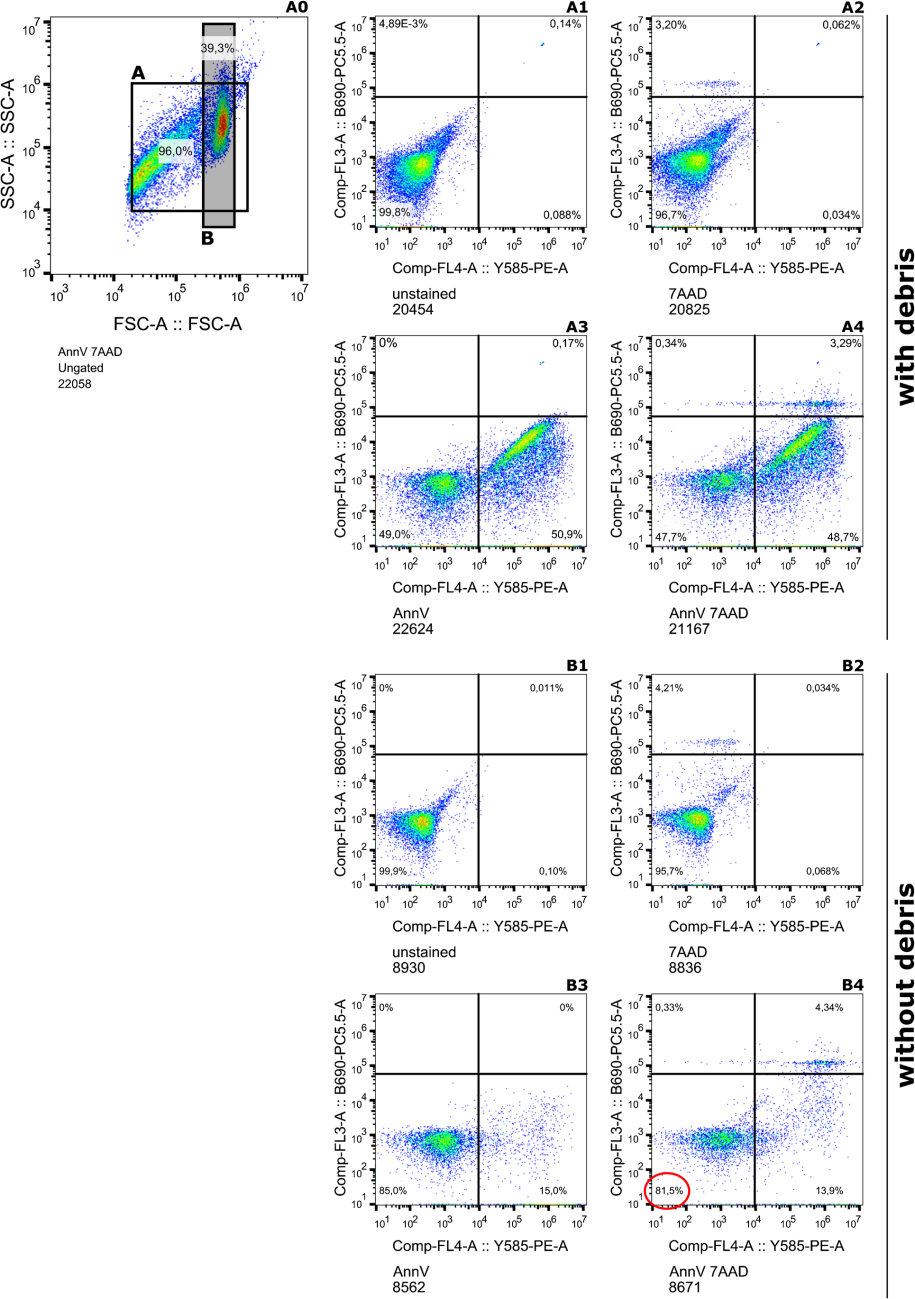
Annexin staining. AnnV/7AAD, annexin V/7-amino-actinmycin; (A0) scattering of the whole sample without staining; **A** (upper 4 graphs) analysis with debris; **B** (bottom 4 graphs) analysis without debris; (A1) 7AAD staining, (A2) combined AnnV + 7AAD staining, (A3) AnnV staining, and (A4) unstained; (B1) 7AAD staining, (B2) combined AnnV + 7AAD staining, (B3) AnnV staining, and (B4) unstained; red circle: marks percentage of cells that are neither shows signals for AnnV or 7AAD staining (81, 5%)

To assess cell viability, isolated cells were stained with Annexin V and 7-amino-actinomycin (7-AAD) to determine the proportions of intact, early apoptotic, and/or physically damaged cells directly after MSC isolation. More than 60% of detected events were regarded as debris resulting from fragmented cells based on forward/side scatter. A population of events comprising around 39% of all events was considered to represent intact cells and was further analyzed. Around 81.5% of intact cells were neither in an early apoptotic state nor physically damaged (Figs.2 and 3). Thus, the results show that the majority of intact cells maintain their viability.

Cells from all donors (*n* = 5) were analyzed via flow cytometry to determine the expression of MSC stemness markers after adhering. The analyzed cells expressed positive markers characteristic for MSCs (CD90, CD105, CD 73) and were negative for markers of other cell populations such as monocytes, leukocytes, or lymphocytes (CD34, CD41b, CD19, CD45, HLA-DR) [29]. Thus, it was shown that MSCs were successfully isolated from donor adipose tissue. Figure 2 displays the flow cytometry results from a representative donor.

#### Cell proliferation and differentiation in response to blood product supplementation

**Fig. 4:**
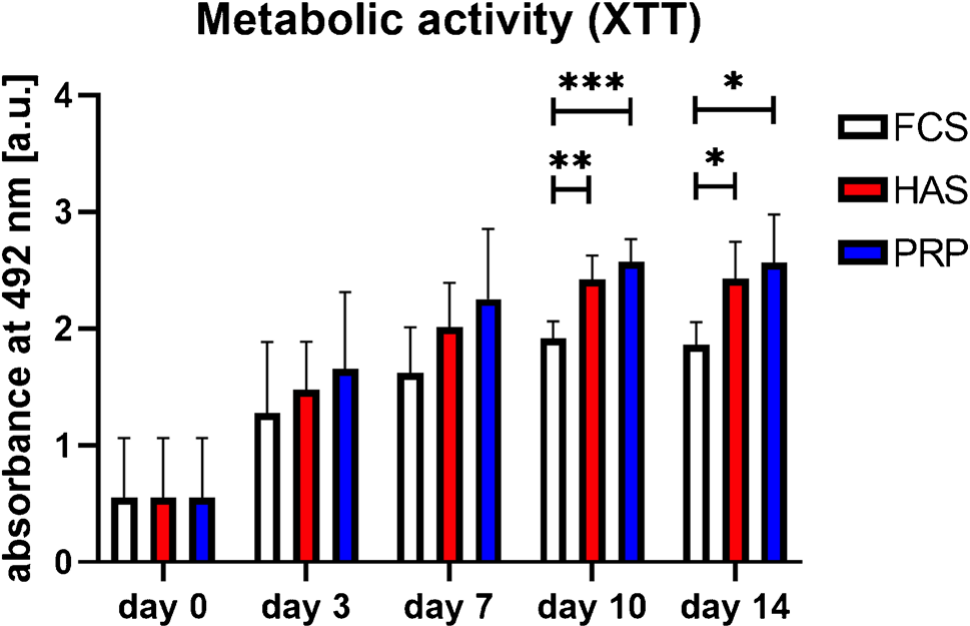
Metabolic activity/viability (FCS, fetal calve serum; HAS, hyperacute serum; PRP, platelet rich plasma; *n* = 5 donors, * = significant. Results are expressed as the mean ± SD. ∗ represents *p* < 0:05, ∗  ∗ represents *p* < 0:01, and ∗  ∗  ∗ represents *p* < 0:001)

An increase in metabolic activity over the entire observation period of 14 days was observed for IFP-MSCs supplemented with either 10% FCS, 10% HAS, or 10% PRP. As determined via one-way ANOVA for each day comparing the different supplementations, significant differences were observed on day 10 (F (2, 12) = 17.24; *p* = 0.0003) and day 14 (F (2, 12) = 6.905; *p* 0.01). FCS-supplemented cells had lower metabolic activity compared to PRP (*p* = 0.0003) or HAS (*p* = 0.0027) supplementation on day 10, as well as on day 14 (*p* = 0.01 and *p* = 0.04, respectively). These results indicate a trophic effect mediated by the human blood products. No significant difference in metabolic activity between HAS and PRP-supplemented cells was observed at any time point (Fig. 4).

#### PCR

**Fig. 5:**
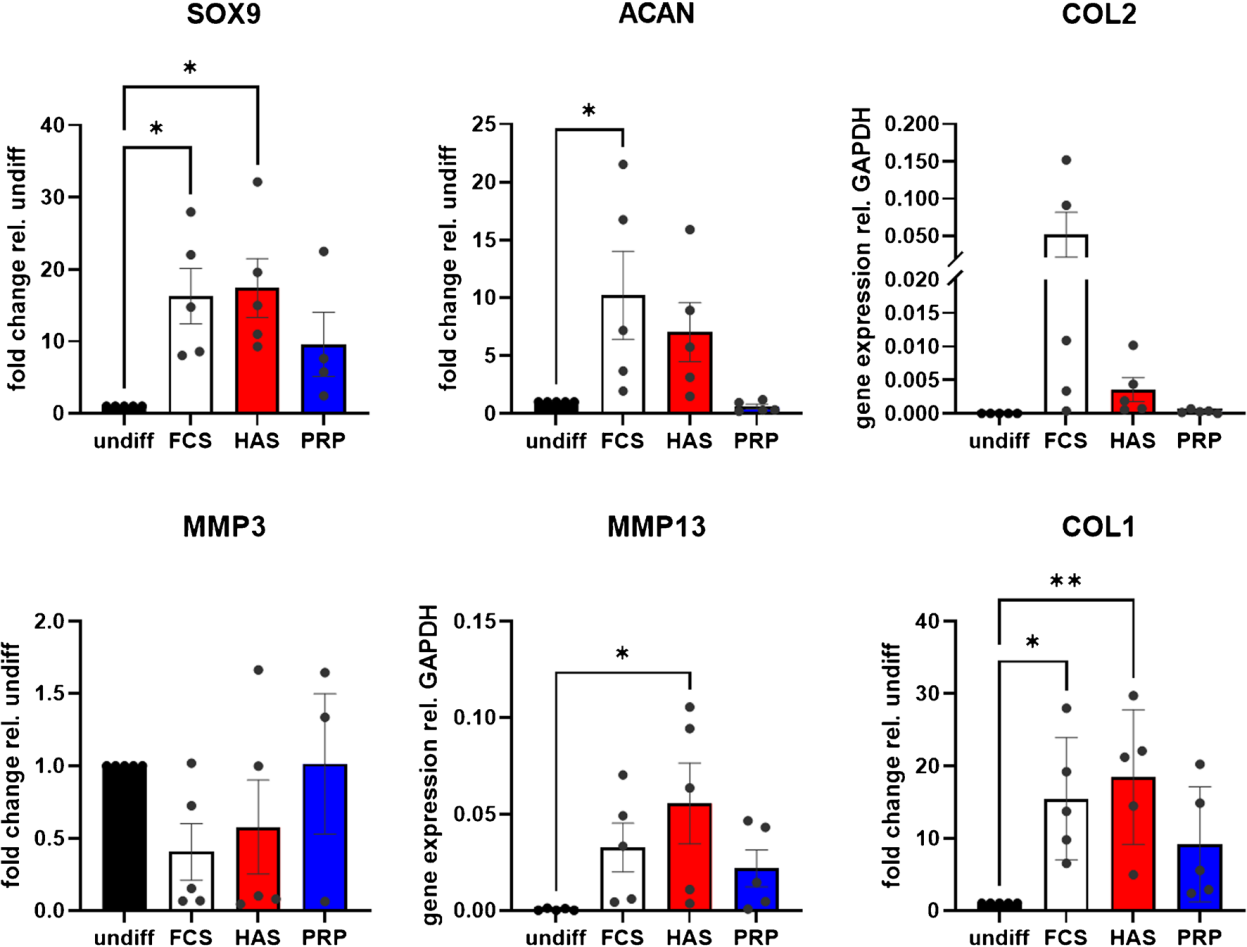
Gene expressions for chondrogenic differentiation experiments. ACAN, aggrecan; COL1, collagen 1A1 gene; COL2, collagen 2 gene; PRP, platelet-rich plasma (blue); FCS, fetal calve serum (white); HAS, hyperacute serum (red); MMP3(/13), matrix metalloproteinase-3(/13) gene; SOX9, SRY-box transcription factor 9. Results are expressed as the mean ± SEM. ∗ indicates a *p* < 0.05 as a significant difference in gene expression within one treatment group. ∗  ∗ represents *p* < 0.01, and ∗  ∗  ∗ represents *p* < 0.001

Gene expression changes were assessed via quantitative PCR in Hoffa MSCs in comparison to cells differentiated to chondrocytes in chondropellets. COL2 and MMP13 were not expressed in undifferentiated cells, therefore, expression relative to GAPDH in chondropellets rather than fold changes was analyzed. Under the exclusion of an outlier from chondropellets differentiated in the presence of CPRP (67.68), SOX9 expression was elevated (F (3,15) = 4.887, *p* = 0.015) in differentiated cells with FCS and HAS resulting in significant upregulation (*p* = 0.017 and 0.011, respectively). Similarly, ACAN expression was elevated (F (3,16) = 4.202, *p* = 0.023) in the presence of FCS supplementation (*p* = 0.031), while HAS supplementation failed statistical significance (*p* = 0.191). Neither COL2 nor MMP3 expression was significantly different under the exclusion of 2 outliers for PRP-supplemented pellets (11.49 and 197.23). Expression of MMP13 was elevated presence of HAS (*p* = 0.024), although ANOVA missed significance (F (3,16) = 3.037, *p* = 0.0596). Finally, COL1 expression significantly increased (F (3,16) = 5.393, *p* = 0.009). Again, the presence of FCS and HAS resulted in significant increase (0.019 and 0.005, respectively) in contrast to PRP supplementation. Comparison of gene expression between chondropellets differentiated in the presence of FCS, HAS, or PRP yielded no significant differences Fig. 5.

#### Histology

**Fig. 6:**
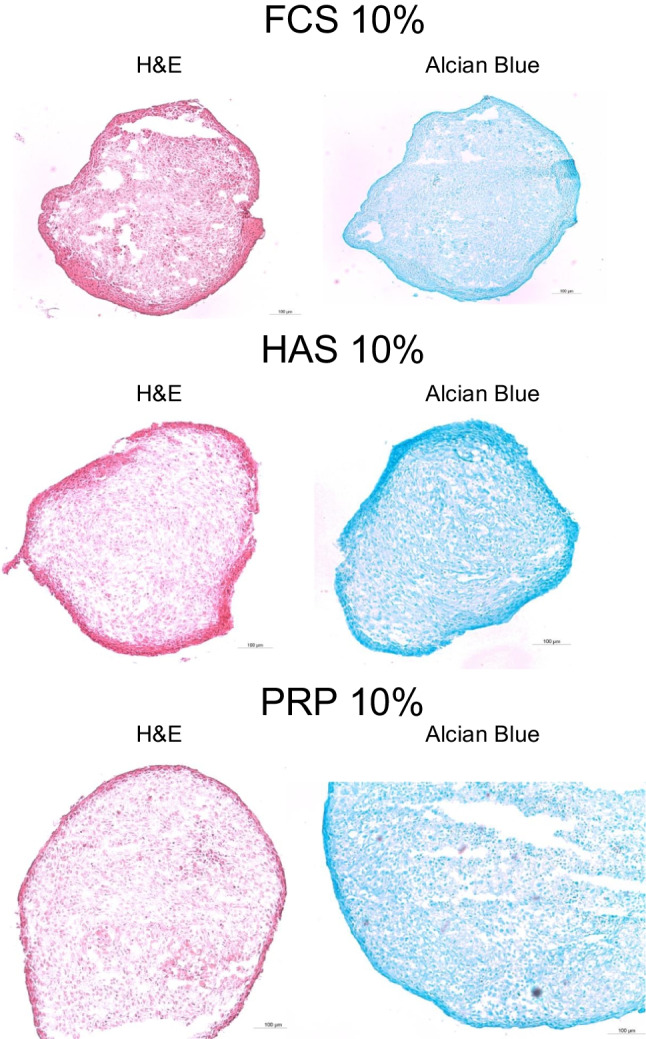
Histology results: HAS, hyperacute Serum; PRP, platelet-rich-plasma, FCS, fetal calve serum; H&E, hematoxylin–eosin staining

IFP-MSCs chondrogenically differentiated in 3D pellet culture in the presence of 10% (v/v) of either FCS, HAS, or CPRP were analyzed via histologic sectioning and Alcian blue staining for deposition of cartilage extracellular matrix. The micrographs shown in Fig. 6 indicate a successful chondrogenic differentiation process in the presence of all blood product supplementations. In a qualitative assessment, a homogenous extracellular matrix organization was shown in all three samples. White spots in between stained tissue are likely artifacts from slicing the samples.

## Discussion

The objective of this work was to evaluate the maintenance of the regenerative potential of IFP-MSCs after arthroscopic harvest and potential enhancing effects of BPs.

The main findings of the presented work are as follows:

Flow cytometry data showed that the isolation protocol worked as expected and IFP-MSCs could be successfully isolated as the stem cell populations expressed typical surface markers [30].

Both blood products increase the metabolic activity and viability, respectively, of IFP-MSCs significantly. No recommendation for a specific BP can be given from those data so far.

With regards to metabolic activity and IFP-MSC viability, respectively, PRP and HAS did not show significant differences in direct comparison. However, a significant increase in metabolic activity in comparison to FCS was shown already on day seven versus day 10 for HAS. Likewise, the significance levels differed with PRP versus FCS having a highly significant increase on day 14 (*p* < 0.001) and HAS versus FCS having a significant increase on day 14 (*p* < 0.05).

MSCs’ chondrogenic differentiation potential was shown to be influenced by BP supplementation resulting in different gene expression levels.

The presented data showed an enhancing effect of BPs upon the gene expression levels after chondrogenic differentiation. HAS was shown to have a significantly higher expression of COL2 and ACAN in comparison to PRP.

Recent literature shows divergent effects of blood products on the chondrogenic differentiation of adipose-derived MSCs [31, 32].

A potential biological explanation may be differing growth factor concentrations [21, 33]. High, non-physiological concentration of growth factors in PRPs as well as the presence of anticoagulants was even shown to have a negative effect on cell viability [34]. In the case of HAS, the “natural” coagulation cascade is not delayed or stopped via an anticoagulant resulting in a blood product containing no platelets and inferior levels of growth factors in comparison to PRP [21].

It may be noted that gene expression levels of associated fold changes vary between analyzed markers.

A possible explanation is the four different stages during chondrogenic differentiation that Xu et al. showed with a specific gene expression profile at each stage [35]. The differentiation protocol applied lasted 21 days. Twenty-one days fall in the “late stage”/stage 4 of Xu et al. stages with dominant expressions of ACAN.

Cells’ viability is also a frequently discussed issue in the literature [36]. Given apoptotic cell yields of up to 50% after liposuction, arthroscopic IFP harvest may appear to be preferable due to viable cell yields beyond 80% as shown in this study [37].

Limiting factors of this study include the in-vitro design and the small tissue quantity harvested with regard to clinically relevant conclusions.

Despite the importance of in vitro data as a driver for translation, the potentially poor comparability to in vivo scenarios is a limiting factor.

A 3D pellet culture was chosen to address this issue because it mimics conditions in physiological cartilage more closely compared to 2D cultures [38, 39].

Another potentially limiting factor for arthroscopic IFP harvest may be the seemingly small tissue quantity. Likewise, the cell yield may appear to be low. However, it was previously shown that smaller MSC cell yields may eventually have more significant clinical effects in comparison with higher cell yields [40]. Moreover, the cell populations counted and analyzed via annexin V staining in the presented work were not only IFP-MSCs but various cells from IFP tissue. However, the tissue used in clinical practice is often fragmented tissue or the “stromal vascular fraction.” In both cases, a variety of cells are present and, thus, re-administered into the joint [41].

Routinely re-applying minced autologous IFP-MSCs in a point-of-care setting, potentially supplemented with blood products, may significantly improve the quality of regenerative knee arthroscopic care.

Depending on the pathologies found during the arthroscopy, the surgeon may decide to follow a tissue engineering-regenerative path for isolated OCLs or a disease-modifying path for OA.

In the first case, seeding MSCs on scaffolds such as fibrin-polyurethan, collagen, or gels for treating OCLs has been shown to be effective [6–8, 42, 43].

In case of more advanced, degenerative lesions and OA, respectively, MSCs were also shown to be beneficial when injected into the joint [3, 44].

## Conclusion

The provided data support the conclusion that arthroscopically harvested IFP-MSCs seem to be a reasonable source of MSCs for orthopaedic use.

Three main general considerations support this proposition:

Firstly, the process of stem cell isolation and potential cultivation was shown to be easier than for chondrocytes [45]. More so, it was shown that chondrocytes tend to de-differentiate in ex vivo cultures [46].

Secondly, the chondrogenic differentiation potential of MSCs in combination with their paracrine effects as well as their engrafting potential is key for cartilage treatments in vivo [47, 48].

Thirdly, this harvesting method allows for “homologous use”—cells from within the joint are being re-injected into the joint. The common practice of re-administering subcutaneous derived MSCs into joints is referred to as “non-homologous use.” Regulatory hurdles are being eased by homologous use [49].

The presented study shows that arthroscopic IFP-MSC harvest leads to likely sufficient tissue quantity and cell yields with maintained cell viability. Likewise, the cell regenerative potential of isolated MSCs is maintained with regard to chondrogenic differentiation. Blood product supplementation further supports the IFP-MSCs viability and their regenerative potential.

## Data Availability

Data available on request due to restrictions, e.g., privacy or ethical. The data presented in this study are available on request from the corresponding author.
